# Incorporating perspectives of self‐advocates and families towards more accessible research of Alzheimer’s disease in Down Syndrome

**DOI:** 10.1002/alz70859_107691

**Published:** 2025-12-26

**Authors:** Sarah Walter, Lauren Ptomey, Ann D Cohen, Michael S. Rafii, Elizabeth Head

**Affiliations:** ^1^ Alzheimer's Therapeutic Research Institute, University of Southern California, San Diego, CA USA; ^2^ University of Kansas Medical Center, Kansas City, KS USA; ^3^ University of Pittsburgh Alzheimer's Disease Research Center (ADRC), Pittsburgh, PA USA; ^4^ Alzheimer's Therapeutic Research Institute, Keck School of Medicine, University of Southern California, San Diego, CA USA; ^5^ University of California, Irvine, Irvine, CA USA

## Abstract

**Background:**

Over 250,000 people live with Down Syndrome (DS) in the United States, with advances in healthcare and support leading to an average life expectance of 63.5 years of age. With increased age, individuals carry a high risk for Alzheimer’s disease (AD); which is now the leading cause of death over the age of 35 years. As clinical trials for dementia treatments and prevention are developed for this population, ensuring accessibility and inclusivity is essential. Gaining insights from self‐advocates with DS and their care partners will help researchers design trials that better meet their needs.

**Method:**

A Research partnership group was formed in 2024, with support from The Alzheimer’s Clinical Trials Consortium ‐ Down Syndrome (ACTC‐DS) and Alzheimer’s Biomarker Consortium Down Syndrome (ABC‐DS). Six self‐advocates have joined the group, along with their care partners and five researchers. The group is founded on the values of collaboration and partnership, guiding its approach to building relationships and sharing lived experiences. The group held five zoom meetings in 2024 to gather feedback from self‐advocates and care partners on three new clinical trials and to address member questions about AD.

**Result:**

The themes that emerged from this partnership group’s feedback will be shared, accompanied by recordings of member perspectives with concrete application for researchers.

Themes include:

(1) The strong motivation to engage in AD research.

(2) The need for diverse and accessible study materials that describe how participants will be supported.

(3) The need for researchers to speak directly to the individual with DS.

(4) The need for research to accommodate high co‐morbidities and busy lives of both self‐advocates and support partners.

(5) Solutions to decrease participant and care partner burden.

(6) The need for frequent research updates to address the dearth of information on aging well with DS.

(7) Involving self‐advocates on research teams can allow meaningful involvement and outcomes most relevant for people with DS.

**Conclusion:**

Involving self‐advocates and families with DS as research partners has provided ACTC‐DS and ABC‐DS with valuable feedback to increase the accessibility of dementia studies. This work will continue to address the health priorities of individuals with DS.

